# Effects of semaglutide on vascular structure and proteomics in high-fat diet-induced obese mice

**DOI:** 10.3389/fendo.2022.995007

**Published:** 2022-11-07

**Authors:** Lin Yue, Shuchun Chen, Qingjuan Ren, Shu Niu, Xiaoyu Pan, Xing Chen, Zelin Li, Xiaoyi Chen

**Affiliations:** ^1^ Department of Internal Medical, Hebei Medical University, Shijiazhuang, China; ^2^ Department of Endocrinology, The Third Hospital of Shijiazhuang, Shijiazhuang, China; ^3^ Department of Internal Medical, Hebei General Hospital, Shijiazhuang, China; ^4^ Department of Endocrinology, Shijiazhuang People’s Hospital, Shijiazhuang, China

**Keywords:** obesity, semaglutide, vascular stiffness, extracellular matrix, proteomics

## Abstract

**Background:**

Obesity is a chronic metabolic disease caused by a combination of genetic and environmental factors. To determine whether semaglutide could improve aortic injury in obese C57BL/6J mice, and further explore its molecular mechanism of action using proteomics.

**Methods:**

24 C57BL/6J male mice were randomly divided into normal diet group (NCD group), high-fat diet group (HFD group) and high-fat diet + semaglutide group (Sema group, semaglutide (30 nmol/kg/d) for 12 weeks). The serum samples were collected from mice to detect blood glucose, insulin and blood lipid concentrations. Aortic stiffness was detected by Doppler pulse wave velocity (PWV). Changes in vascular structure were detected by HE, masson, EVG staining and electron microscopy. The aorta-related protein expression profiles were detected by proteomic techniques, and proteins with potential molecular mechanisms were identified.

**Results:**

Semaglutide could reduce body weight, the concentrations of blood glucose, total cholesterol (TC), triglycerides (TG), lipoprotein cholesterol (LDL-C), and reduce the aortic PWV and ameliorate vascular damage in obese mice. The results of proteomic analysis showed there were 537 up-regulated differentially expressed proteins (DEPs) and 322 down-regulated DEPs in NCD/HFD group, 251 up-regulated DEPs and 237 down-regulated proteins in HFD/Sema group. There were a total of 25 meaningful overlapping DEPs in the NCD/HFD and HFD/Sema groups. GO enrichment analysis of overlapping DEPs found that these differential proteins were mainly located in the signaling pathways of the extracellular matrix. The most obvious changes of extracellular matrix associated proteins in the three experimental groups were Coll5a1, Lama4, Sparc.

**Conclusion:**

Semaglutide may protect vascular structure and improve endothelial permeability by reducing the levels of Coll5a1, Lama4, Sparc in extracellular matrix, so as to improve vascular function and achieve vascular protection.

## Introduction

Obesity in adults is a chronic metabolic disease caused by a combination of genetic and environmental factors. The worldwide prevalence of overweight and obesity is high and increasing (1). Obesity can cause multiple cardiovascular complications and increase the risk of chronic diseases. On the one hand, obesity will produce a variety of structural and functional changes in the macrovascular system in population. On the other hand, obesity also indirectly affects the macrovascular system through its effects on related comorbidities (such as dyslipidemia, hypertension, insulin resistance, etc.) (2). It has been shown that fed high-fat diet (HFD) developed obesity associated with impaired endothelium-dependent vasodilation and increased arterial wall thickness in mice (3). Under obesity stimulation, vascular endothelial cells increase cell adhesion and procoagulant phenotypes and barrier function defects through NF-κB signaling molecules, reducing NO bioavailability in mammals (4–6). Also under the effects of obesity and insulin resistance, the extracellular matrix (ECM) was remodeled, and its composition and structure were altered. Synthesis of ECM proteins were increased such as fibronectin and collagen, leading to decreased vascular compliance and vascular stiffness (7–11).

Glucagon-like peptide-1 receptor agonists (GLP-1RA) enhance insulin secretion and lower blood glucose in a glucose concentration-dependent manner by activating GLP-1 receptors. Semaglutide is the latest long-acting GLP-1R agonist. Semaglutide has both hypoglycemic and weight loss effects, and further optimizes high-affinity albumin binding, extending its human plasma half-life to 160 hours (12). In the Semaglutide in Subjects with Type 2 Diabetes (SUSTAIN-6) clinical trial, a reduction in cardiovascular disease risk and a significant improvement in cardiovascular outcomes were observed in patients with type 2 diabetes treated with semaglutide (13). Most studies on GLP-1R agonists have focused on the protective mechanisms of diabetes-induced atherosclerosis.

However, there were few studies on the protective effect of obesity-induced early vascular injury, and the molecular mechanism of drug action was lacking. Diabetes and obesity shared common pathogenic mechanisms that led to vascular damage. Therefore we hypothesized that semaglutide was also protective against obesity-induced vascular damage. To test this hypothesized, we established an obese mice model to examine changes in vascular structure and function. Proteomics technology was used to observe the changes of aortic protein expression profiles of obese mice before and after semaglutide intervention for the first time. And further to explore the molecular mechanism of the protective effect of semaglutide on vascular injury in obese mice.

## Materials and methods

### Animal experiments

6-week-old C57BL/6 male mice were housed under standard laboratory conditions (room temperature at 22 ± 2°C, indoor humidity at 55 ± 10%) with a 12-hour light and 12-hour dark cycle per day, and water ad libitum. 24 C57BL/6J male mice were randomly divided into normal diet group (NCD group), high-fat diet group (HFD group) and high-fat diet + semaglutide group (Sema group). NCD group: Mice were given a control diet (4% fat, 20% protein, carbohydrates, total calories 34.8 kcal/100 g). HFD group: Mice were given a high-fat diet (60% fat, 20% protein, 20% carbohydrate, total calories 524 kcal/100g). Sema group: Mice were given a high-fat diet only for 12 weeks, then daily received subcutaneous injection of semaglutide (novo nordisk A/S, Denmark) 30 nmol/kg/d and given a high-fat diet for 12 weeks simultaneously. After experiment, all mice were bled from the eyeballs. Blood was coagulated at 4°C for 30 min, centrifuged at 3,000 × g for 15 min, and serum supernatant was collected and stored at -80°C. The aorta was removed by thoracotomy, quickly placed in liquid nitrogen, and cryopreserved at -80°C.

### Intraperitoneal glucose tolerance test

Mice were fasted for 16 h, then intraperitoneally injected with 2 mg/g glucose. Blood was collected from the tail vein of the mice before and 15, 30, 60, 90, and 120 min after injection of glucose, and the whole blood glucose concentration was measured using a Roche glucometer.

### Assessment of plasma levels

Determination of mouse insulin levels using Elabscience mouse insulin enzyme-linked immunosorbent assay kit (Wuhan Elarite Biotechnology Co., Ltd.) (14). Serum total cholesterol (TC), triglycerides (TG), lipoprotein cholesterol (LDL-C) and high-density lipoprotein cholesterol (HDL-C) levels were determined by COD-PAP, GPO-PAP, and commercially available kits (Nanjing Jiancheng) configured by microplate method, respectively. All assays were detected using an automatic microplate reader (VERSAmax, America) and analyzed using SOFTmax PRO 4.3LS software (15).

### Measurement of aortic stiffness by *in vivo* pulse wave velocity

Under isoflurane anesthesia (100%, oxygen flow 1.75%), velocity waveforms were acquired at the aortic arch using a Vevo2100 high-resolution small animal ultrasound machine (VisualSonics, Canada) (11). Measurements were then taken immediately at the descending aorta 35 mm distal to the aortic arch. It can be calculated by using the following formula: D (meters)/t (seconds). It was the time difference between two fixed distances along the aorta by measuring the pressure pulse and D was the distance between the two fixed locations along the aorta. *In vivo* aortic stiffness was assessed by pulse wave velocity (PWV).

### Morphological and histological evaluation

The aorta of each mouse was isolated, and about 3 mm of the aortic arch was removed, fixed in 4% paraformaldehyde, dehydrated, embedded in paraffin, and then sliced ​​with a microtome. Hematoxylin-eosin (HE) staining, Masson staining and Elastica van Gieson (EVG) staining were performed, respectively. The region of interest was photographed using an Eclipse Ci-L photographic microscope (Nikon, Japan). Image-Pro Plus 6.0 analysis software (Media Cybemetics, USA) was used to measure the area and perimeter of the vascular media, and the area and perimeter of the vascular lumen in each slice, respectively. Calculated media thickness = (media area - vessel lumen area) * 2/(media circumference + vessel lumen circumference). Use the Eclipse Ci-L photo microscope to image the target area at 200 times, and measure the pixel area of ​​elastic fibers and the pixel area of ​​blood vessels in each image. Calculate the percentage of elastic fiber area by elastic fiber pixel area/visual field pixel area*100. Calculate the collagen area and tissue area in the measurement area, and calculate the proportion of collagen area.

### Ultrastructural analysis by transmission electron microscopy

A 1 mm thoracic aortic ring was placed in electron microscope fixative (Servicebio, G1102) (16). Samples were then embedded, cut into 50 nm sections, and stained with 3% uranyl acetate and lead citrate. Transmission Electron Microscopy (TEM) images were collected on a transmission electron microscope (HT7700, Hitachi, Japan).

### Protein extraction and TMT labeling

The aorta of mice was grouded and added in SDT (4%SDS, 100 mM Tris-HCl, 1 mM DTT, pH7.6) buffer for lysis. Detergents, DTT and other low molecular weight components were removed from protein samples with UA buffer (8 M Urea, 150 mM Tris-HCl pH 8.0) (17). The solution was alkylated with 100 μl iodoacetamide (100 mM IAA in UA buffer) for 30 min at room temperature in the dark. Finally, the protein were digested with 4 μg trypsin (Promega) overnight at 37°C, and the resulting peptides were collected. According to the manufacturer’s instructions (Thermo Scientific), 100 μg peptide mixture of each sample was labeled using TMT reagent.

### Liquid chromatography-mass spectrometry mass spectrometry analysis

The samples were dissolved in buffer A (0.1% Formic acid) and B (84% acetonitrile and 0.1% Formic acid), before separated by the C18-reversed phase analytical column (Thermo Scientific Easy Column, 10 cm long, 75 μm inner diameter, 3μm resin) (18). Each peptides was analyzed by a Q Exactive mass spectrometer (Thermo Scientific) that was coupled to Easy nLC for 60/90 min. Using a data-dependent top10 method for beam-type CID fragmentation (HCD) to obtained MS data, and using the MASCOT engine (Matrix Science, London, UK; version 2.2) embedded into Proteome Discoverer 1.4 software for identification and quantitation analysis.

### Bioinformatic analysis

The Cluster 3.0 (http://bonsai.hgc.jp/~mdehoon/software/cluster/software.htm) and Java Treeview software (http://jtreeview.sourceforge.net) were used to analyze hierarchical clustering (19). CELLO (http://cello.life.nctu.edu.tw/) and interproscan software were used to predict protein subcellular localization and identify protein domain signatures. The NCBI BLAST+ client software (ncbi-blast-2.2.28+-win32.exe) and InterProScan were used to find homologue sequences and annotate. Kyoto Encyclopedia of Genes and Genomes (KEGG) database were used to annotate protein pathways and classify these pathways.

### Data analysis

All statistical analyses were performed using the GraphPad Prism 8.0 software. The experimental data are expressed as mean ± SD the standard error of the mean. The multiple comparisons were analyzed using one-way ANOVA. Statistically significance was accepted at P<0.05 level (*),P<0.01 level (**).

## Results

### Semaglutide reduces body weight, improves blood sugar, blood lipids, and insulin levels in mice

**Figure 1:**
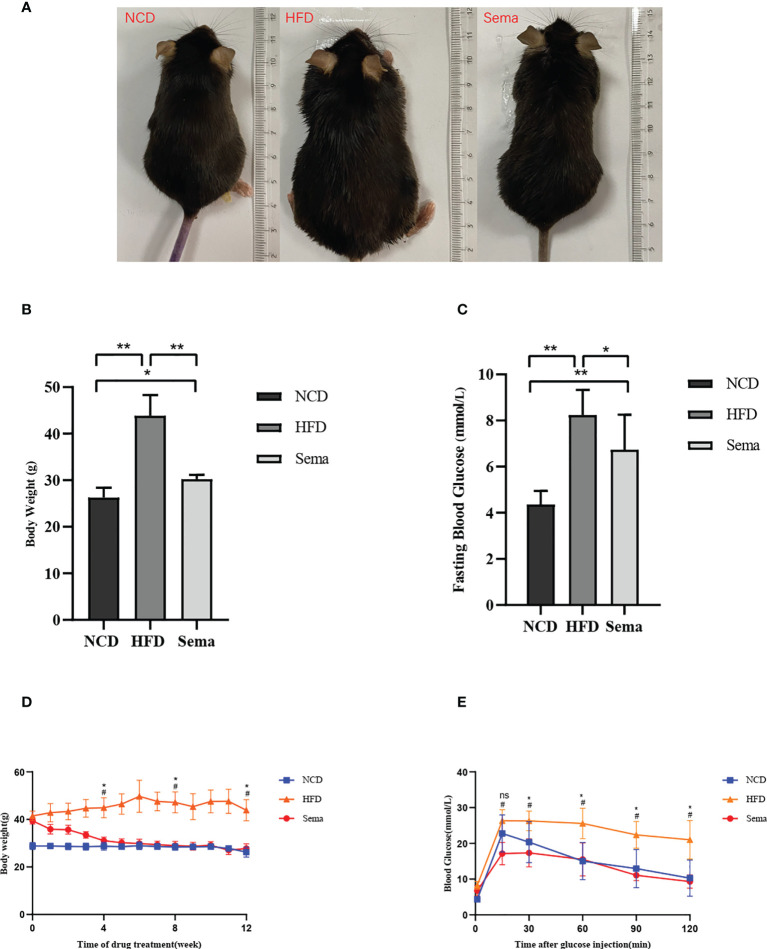
**(A)** The body weight and appearance of mice in three groups were changed. **(B)** The body weight changes of the three groups. **(C)** Blood glucose changes in three groups. **(D)** Body weight change trend of mice in three groups from 4 to 12 weeks. **(E)** Blood glucose change trend of mice in three groups from 4 to 12 weeks. Results are expressed as mean ± SD the standard error of the mean. (n=8). * means P <0.05; ** means P <0.01; ^#^ means HFD vs Sema; ns means not significant.

**Figure 2:**
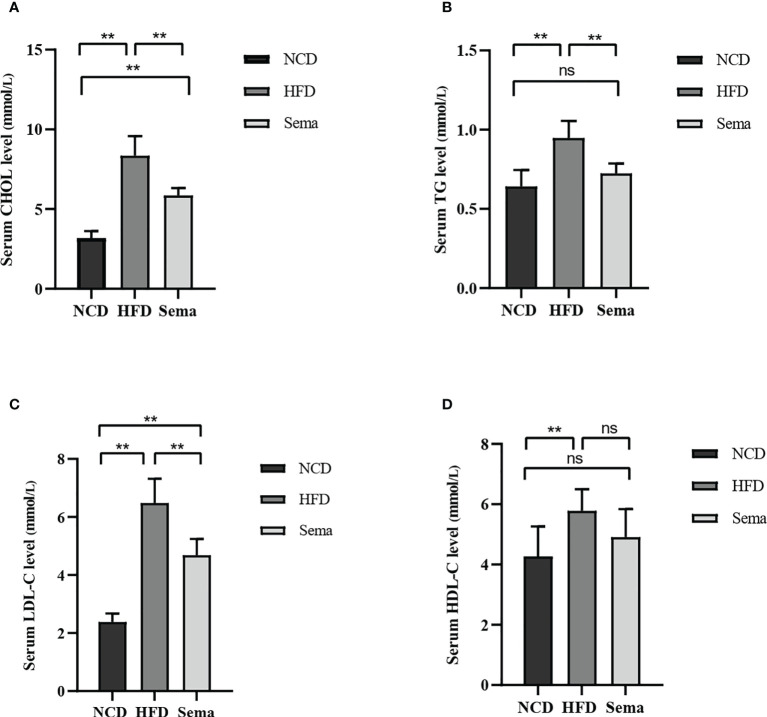
**(A)** CHOL levels in three groups of mice. **(B)** TG levels in three groups of mice. **(C)** LDL-C levels in three groups of mice. **(D)** HDL-C levels in three groups of mice. Results are expressed as mean ± SD the standard error of the mean. (n=8). ** means P < 0.01; ns means not significant.

The body weight changes of the three groups were compared at the end of the experiment **(** **Figure 1A** **)**. The results showed that the weight of mice in HFD group was significantly higher than that in NCD group, and the hair of mice was shiny (P<0.05). The weight of mice in Sema group was significant lower than that in HFD group (P<0.05, **Figure 1B**). During the 4th to 12th week of semaglutide intervention, the weight of mice in Sema group was not statistically different from that in NCD group (**Figure 1D**). High-fat diet could increase fasting plasma glucose in mice. After semaglutide intervention, fasting plasma glucose in mice and HFD group has a downward trend, but there was significant statistical difference (**Figure 1C**). After 15 min, 30 min, 60 min, 90 min, and 120 min of glucose stimulation, the blood glucose in Sema group was significantly lower than that in the HFD group (**Figure 1E**), which indicating that semaglutide had more significant control of postprandial blood glucose. Furthermore, compared with NCD group, the levels of TC, TG, LDL-C, and HDL-C were significantly increased in HFD group **(** **Figures 2A-D** **)**. Compared with HFD group, TC, TG and LDL were significantly decreased after semaglutide treatment, but HDL-C had no significant change (P<0.05).

### Semaglutide inhibits high-fat diet-induced changes in aortic tissue structure (n=3)

**Figure 3:**
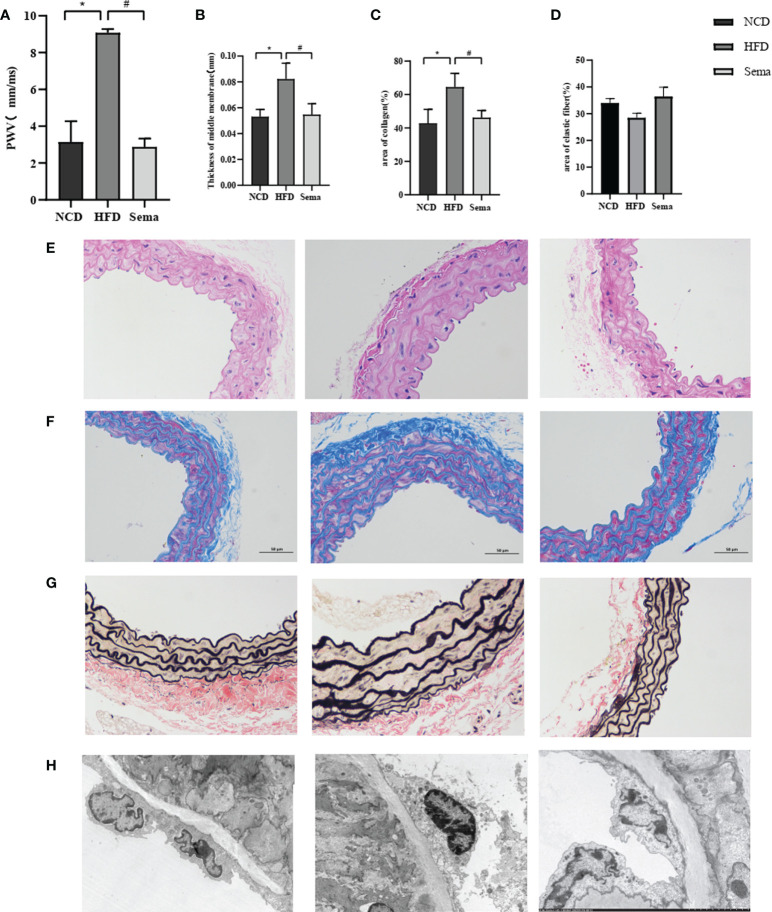
**(A)** PWV of aorta of three groups of mice. **(B)** The thickness of the blood vessel walls of aorta of three groups of mice. **(C)** Collagen levels of aorta of three groups of mice. **(D)** elastin fibers of aorta of three groups of mice. **(E)** The thickness of the blood vessel walls of aorta of three groups of mice in HE. **(F)** The thickness of the blood vessel walls of aorta of three groups of mice in masson staining. **(G)** Collagen levels of aorta of three groups of mice in EVG staining. **(H)** elastin fibers of aorta of three groups of mice in TEM. Results are expressed as mean ± SD the standard error of the mean. (n=3). * means NCD vs HFD; ^#^ means HFD vs Sema.

Compared with NCD group, aortic PWV was significantly greater in HFD group (P<0.05), indicating increased aortic stiffness. Compared with HFD group, the aortic PWV of mice in the Sema group was decreased after semaglutide treatment (P<0.05), indicating that semaglutide reduced aortic stiffness **(** **Figures 3A-D** **)**. HFD mice had thicker blood vessel walls and more extracellular matrix than control mice. However, the thickness of blood vessel wall and extracellular matrix were significantly reduced in mice treated with semaglutide **(** **Figure 3E** **)**. Masson staining showed Collagen fibers appear blue, muscle cells appear red, and overall collagen levels increase in the HFD group, Collagen arrangement disorder, wall thickening. After treatment with semaglutide, the collagen level decreased significantly, the collagen was arranged in parallel, and the tube wall became thinner **(** **Figure 3F** **)**. EVG staining showed that the elastic fibers were blue and black, and the elastic fibers proliferated, cracked and disintegrated in the HFD group. Semaglutide treatment attenuated elastin damage **(** **Figure 3G** **)**. These results showed that semaglutide could reverse vascular remodeling.

### Semaglutide prevents high-fat diet-induced endothelial cell ultrastructural changes

Aortic endothelial cell were elongated and tightly adhered to the internal elastic lamina in NCD group. In HFD group, the aortic endothelial cells were swollen, less adherent, slightly separated from the internal elastic lamina, and the intercellular tight junctions disappeared. Aortic endothelial cell swelling disappeared and adhered tightly to the internal elastic lamina after semaglutide treatment **(** **Figure 3H** **)**.

### Quantification and identification of the aortic proteome by Tandem Mass Tags analysis

**Figure 4:**
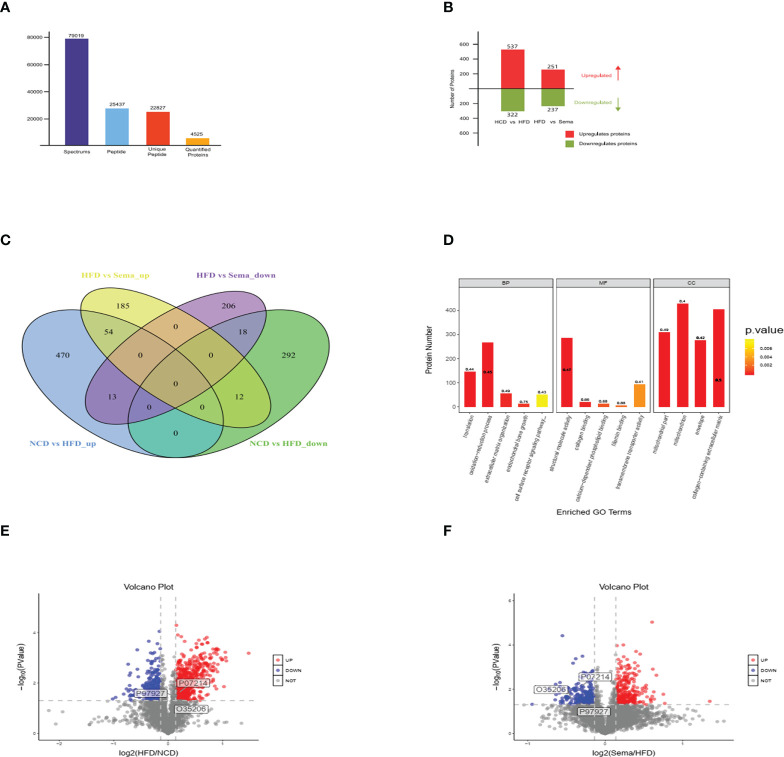
**(A)** LC-MS/MS Mass spectrometry (MS) analysis revealed in total 77,1849 spectrum proteins, 7,9019 matched-spectrum proteins, 2,5437 peptides, 22827 unique peptides, 4532 identified proteins and 4525 quantifiable proteins. **(B)** The number of up-regulated and down-regulated proteins was compared between the two groups. **(C)** The number of up-regulated and down-regulated proteins was compared between the two groups in Venn diagram. **(D-F)** The volcano plot of DEPs, Significantly down-regulated proteins are marked in blue, significantly up-regulated proteins are marked in red, and indifferent proteins are marked in grey. (n=3).

LC-MS/MS Mass spectrometry (MS) analysis revealed in total 77,1849 spectrum proteins, 7,9019 matched-spectrum proteins, 2,5437 peptides, 22827 unique peptides, 4532 identified proteins and 4525 quantifiable proteins **(** **Figure 4A** **)**. According to the literature (20), the proteins that showed P value<0.05 and a Fold Change (FC) >1.1 were defined a differentially express proteins. After data analysis, the change threshold of DEPs was determined. An increase in expression greater than 1.1-fold was considered up-regulation, while a decrease in expression of greater than 1.1-fold was considered down-regulation. There were 859 DEPs in the NCD/HFD groups, containing 537 up-regulated proteins and 322 down-regulated proteins. There were 488 DEPs in HFD/Sema group, containing 251 up-regulated proteins and 237 down-regulated proteins **(** **Figure 4B** **)**. The volcano plot of DEPs was shown in **Figures 4E, F**. There were 25 overlapping DEPs between HFD/Sema and NCD/HFD in Venn diagram, including 12 up-regulated and 13 down-regulated proteins **(** **Figure 4C** **)**.

### Protein domain analysis of differential proteins

**Figure 5:**
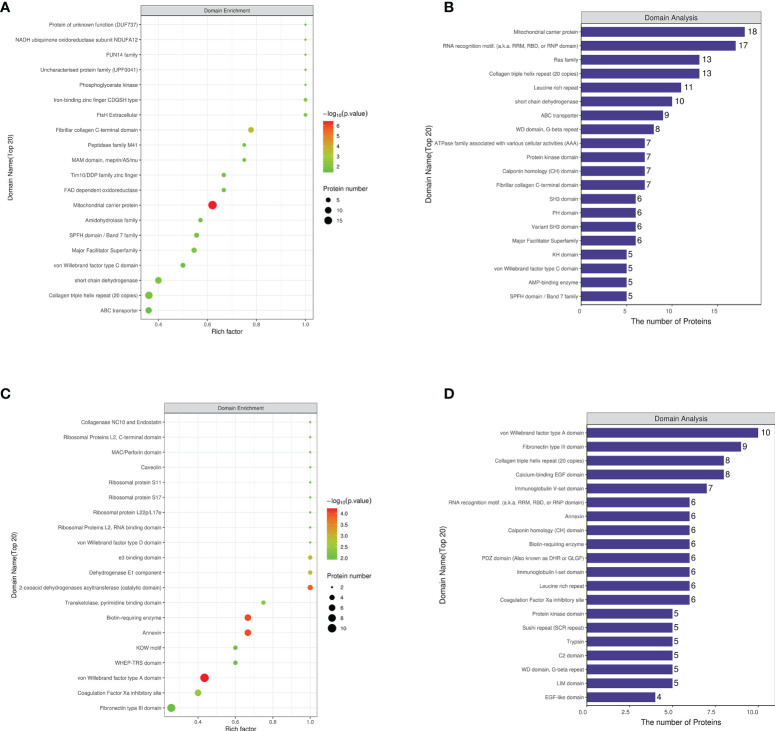
**(A)** The first 20 regions where the enrichment factor accumulates in NCD/HFD. **(B)** The number of proteins in the first 20 regions where enrichment factors are concentrated in NCD/HFD. **(C)** The first 20 regions where the enrichment factor accumulates in HFD/Sema. **(D)** The number of proteins in the first 20 regions where enrichment factors are concentrated in HFD/Sema. (n=3).

The differential protein domains of the NCD/HFD group were mainly concentrated in the mitocheondrial carrier protein, fibrillar collagen C-terminal and Collagen triple helix repeat (20 copies). The differential protein domains of the HFD/Sema group were mainly concentrated in Biotin-requiring enzyme, Annexin, von Willebrand factor type A domain, 2-oxoacid dehydrogenases acyltransferase (catalytic domain), Fibronectin type III domain **(** **Figures 5A-D** **)**.

### Subcellular localization analysis of differential proteins

**Figure 6:**
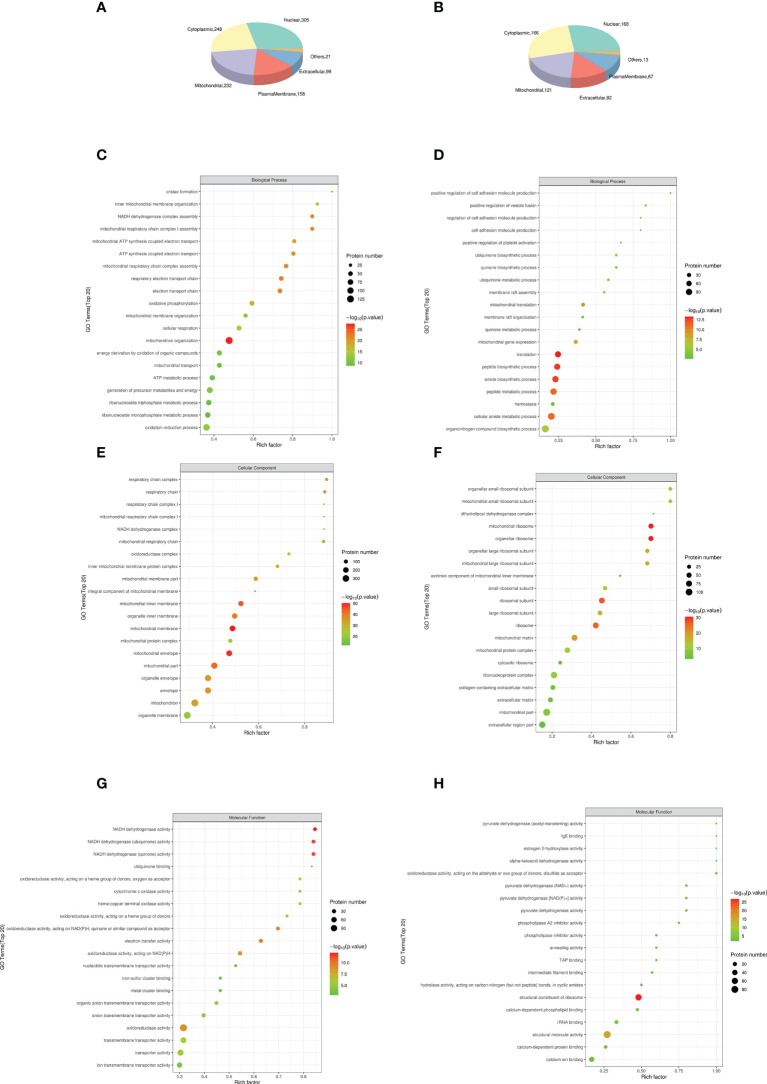
The top 15 most significantly enriched GO terms enriched in DEPs of KD. **(A, B)** Subcellular localization analysis of differential proteins. **(C)** Biological process (BP) in NCD/HFD. **(D)** Biological process (BP) in HFD/Sema. **(E)** Cellular component (CC) in NCD/HFD. **(F)** Cellular component (CC) in HFD/Sema. **(G)** Molecular function (MF) in NCD/HFD. **(H)** Molecular function (MF) in HFD/Sema. (n=3).

DEP was mainly located in nuclear (28.69% in NCD/HFD, 27.23% in HFD/Sema). The remaining DEPs were mainly located in cytoplasmic (23.33% in NCD/HFD, 26.9% in HFD/Sema), mitochondrial (21.83% in NCD/HFD, 19.61% in HFD/Sema) extracellular (14.86% in NCD/HFD, 13.29% in HFD/Sema) and plasma membrane (9.31% in NCD/HFD, 10.86% in HFD/Sema; **Figures 6A, B**). The DEPs between HFD/NCD and HFD/Sema (**Table 1**).

**Table 1:** The DEPs between HFD/NCD and HFD/Sema.

Accession	Protein Name	Gene Name	HFD/NCD	HFD/Sema
P04918	Serum amyloid A-3 protein	Saa3	0.8213	1.4541
Q99P87	Resistin	Retn	0.6179	1.3057
P01630	Ig kappa chain V-II region 7S34.1		0.8622	1.2510
P05555	Integrin alpha-M	Itgam	0.8352	1.2298
P97427	Dihydropyrimidinase-related protein 1	Crmp1	0.8434	1.2182
P20491	High affinity immunoglobulin epsilon receptor subunit gamma	Fcer1g	0.8462	1.2101
P28663	Beta-soluble NSF attachment protein	Napb	0.8609	1.1740
P70349	Adenosine 5’-monophosphoramidase HINT1	Hint1	0.8068	1.1636
O08759	Ubiquitin-protein ligase E3A	Ube3a	0.8444	1.1474
Q61584	Fragile X mental retardation syndrome-related protein 1	Fxr1	0.7720	1.1228
Q3TXS7	26S proteasome non-ATPase regulatory subunit 1	Psmd1	0.8788	1.1211
Q8R0G9	Nuclear pore complex protein Nup133	Nup133	0.8849	1.1136
P17710	Hexokinase-1	Hk1	1.1147	0.9062
Q91VK4	Integral membrane protein 2C	Itm2c	1.1441	0.8824
Q91WP0	Mannan-binding lectin serine protease 2	Masp2	1.1178	0.8794
Q921R8	Solute carrier family 41 member 3	Slc41a3	1.2734	0.8395
Q8BH64	EH domain-containing protein 2	Ehd2	1.2079	0.8125
O35343	Importin subunit alpha-3	Kpna4	1.2187	0.8088
P07214	SPARC	Sparc	1.1517	0.8078
P97927	Laminin subunit alpha-4	Lama4	1.1002	0.7982
Q4ZJN1	Complement C1q and tumor necrosis factor-related protein 9	C1qtnf9	1.2271	0.7886
Q8C838	Trafficking regulator of GLUT4 1	Trarg1	1.2579	0.7858
P14824	Annexin A6	Anxa6	1.1354	0.7731
O35206	Collagen alpha-1(XV) chain	Col15a1	1.1298	0.7433
P97737	Growth/differentiation factor 10	Gdf10	1.1761	0.6814

### GO analysis of differential proteins

Results obtained according to Gene Ontology GO functional enrichment analysis. In the Biological process (BP) analysis, the functions of the NCD/HFD group mainly involved the mitochondrial respiratory electron chain and ATP synthesis. The functions of the HFD/Sema group mainly involved the mitochondrial translation, peptide and amide biosynthetic and metabolic process. In the Molecular Function (MF) analysis, the two groups of differential proteins mainly have activity and binding functions. In the Cellular Component (CC) analysis, the most abundant structural component was mitochondrial in the two comparison groups. Through the GO enrichment analysis of overlapping DEP, it was found that the extracellular matrix was involved in the enrichment analysis of BP, MF and CC. We speculated that semaglutide ameliorates vascular injury by affecting the extracellular matrix. The expression levels of three proteins related to extracellular matrix, Coll5a1, Lama4 and Sparc, were significantly different (**Figures 6C–H**).

## Discussion

In recent decades, with the increasement in the obese population, obesity-related diseases such as type 2 diabetes and cardiovascular diseases have become a considerable threat to people’s health. Bunbupha et al. found that rats fed HFD for 16 weeks exhibited obesity and metabolic disturbances, as well as vascular and renal dysfunction (11). HFD caused obesity, metaboic disturbances, hypertension and concomitant vascular and renal impairment and morphological changes (3, 21). Thomas et al. showed that weekly semaglutide injections resulted in significant weight loss over 68 weeks in overweight or obese adults (22). Consistent with the above findings, Richard et al. showed a significant reduction in body weight at 26 weeks with oral semaglutide compared with subcutaneous liraglutide (23). Li et al. found that oral semaglutide in diabetic patients did not significantly increase cardiovascular risk and all-cause mortality (24). A double-blind clinical trial conducted by Bo et al. found that semaglutide was more optimized for blood glucose improvement and weight loss than sitagliptin (25). In this study, we established an obese mouse model. Mice of Sema group fed a high-fat diet for 12 weeks then lost significant body weight after semaglutide injection. Mice of HFD group fed a high-fat diet for 24weeks. These results indicated that semaglutide could reduce body weight in mice. Tina et al. found that oral sema in type 2 diabetic patients could improve fasting blood glucose, glycosylated hemoglobin and body weight (26). Consistent with this study, our results found that a high-fat diet increased fasting blood glucose in mice. Fasting blood glucose was significantly decreased after semaglutide intervention. Meanwhile, we found that a high-fat diet significantly increased postprandial blood glucose elevation. The postprandial blood glucose was reduced with semaglutide intervention and returned to normal blood glucose levels in mice. TC, TG, LDL-c are consistent with the above results. Interestingly, a high-fat diet was found to increase HDL-C in our trial, but there was no significant difference after semaglutide intervention. Therefore, the results of this study allow elaborating a conclusion projecting potential treatments in patients with obesity.

PWV measurement was considered the gold standard for assessing vascular stiffness and has independent predictive value for all-cause and cardiovascular mortality (27, 28). We observed that semaglutide reduced the elevation of PWV in mice due to a high-fat diet. Histopathological analysis showed that the aortic walls of obese mice had higher vessel wall thickness, elastic fiber destruction, and a higher degree of collagen accumulation. Treatment with semaglutide ameliorated these changes and reduced PWV. Endothelial dysfunction is an early indicator of atherosclerosis. Electron microscopy results showed that the aortic endothelial cells of obese mice swelled, pyknosis, separated from the inner elastic membrane, and the tight junctions between cells disappeared. Our study showed that semaglutide intervention reverses obesity-related endothelial cell pyknosis and loss of tight junctions.

Changes in vascular characteristics in obese mice suggest that semaglutide has the potential to improve aortic function and structural changes in obese mice. To investigate the molecular mechanism, we applied the proteomic approach of TMT to analyze a total of 4532 proteins quantifiable in the three experimental groups. There were 859 differentially expressed proteins in the NCD/HFD group and 488 differentially expressed proteins in the HFD/Sema group, with a total of 25 significant overlapping differentially expressed proteins. Subsequent GO enrichment analysis showed overlapping DEPs. DEPs are significantly enriched in redox, translation, extracellular matrix. Changes in extracellular matrix may be related to the regulatory effect of semaglutide on aortic injury in obese mice. GO analysis showed that Sema was involved in mitochondrial activity changes in both groups. Therefore, we hypothesized that Sema was involved in the process of mitochondrial energy release to compensate for the damage caused by high-fat diet.

ECM is a dynamic three-dimensional network of macromolecules that provided scaffolding and structural support for blood vessels., And exchange information with cells to regulate the physiological function of blood vessels. The ECM is mainly composed of structural proteins such as basement membrane (BM), collagen, elastin, osteopontin (OPN), and non-structural matrix cellular proteins such as cysteine-rich (Sparc) (29). Changes in ECM composition and structure can lead to reduced vascular compliance, vascular stiffness, and functional changes affecting cell migration and proliferation. Obesity affected the structure of vascular extracellular matrix and cell signaling. On the one hand, obesity can change the composition of extracellular matrix and increase the deposition and cross-linking of vascular collagen (28). On the other hand, the extracellular matrix can regulate smooth muscle cell proliferation, migration and endothelial cell function *via* integrins (30, 31). Among the overlapping differential proteins, we found three proteins closely related to the extracellular matrix, Coll5a1, Lama4, Sparc. Coll5a1, a non-fibrillar member of the collagen superfamily, contains proteoglycan-like chondroitin sulfate side chains (32). Evidence suggested that proteoglycans from the side chain of chondroitin sulfate can bind apoB100 and LDL and promote the development of early arterial lesions (33). A recent study also found that specific Col15a1 knockout mice fed a Western diet for 18 weeks resulted in a dramatic 82% reduction in lesional fibrous collagen content and an increase in carotid artery elasticity (34). Obesity can cause vascular smooth muscle cell proliferation, migration, and arterial stiffness. The application of semaglutide may reduce the proliferation of SMCS by reducing Coll5a1, reduce the binding to LDL, and delay the development of arterial lesions.

Laminin Lama4 is a family of heterotrimeric proteins composed of three polypeptides. Previous studies have reported a role for Lama4 in regulating lipolysis, angiogenesis and even immune inflammation (35–38). Recent studies have demonstrated that Lama4 affects endothelial cell tightness, increases leukocyte extravasation, and promotes monocyte-to-macrophage differentiation (38). Therefore, we speculated that semaglutide improved endothelial cell tightness by reducing Lama4, affected high-fat diet-induced macrophage infiltration, reduced leukocyte migration ability, and alleviated vascular inflammation. Sparc is an ECM-associated protein. Studies have shown that Sparc can disrupt endothelial cell junctions, increase endothelial permeability, and promote leukocyte extravasation and adhesion by activating VCAM1 on endothelial cells (39, 40). There was a study showed that oxLDL-inducible osteonectin expression promotes the development of vascular calcification (41). The high-fat diet-elevated Sparc observed in our study was decreased by semaglutide, with concomitant improvement in vascular damage. We believed that semaglutide can improve endothelial permeability by regulating VCAM1 on endothelial cells *via* Sparc.

There was an observational study of semaglutide on aortic sclerosis in ApoE-/- and Ldlr-/- mice (42). However, there were no protective studies on early injury of the aorta in obese mice. In this experiment, proteomics technology was used for the first time to explore the target of semaglutide from the molecular mechanism of action. There were also some limitations throughout the experiment: Failure to combine HDL functional assays to determine HDL changes, and to combine multi-omics to better interpret drug targets, avoid post-translational modifications and the effects of protein instability. The changes in proteins highlighted in the text (such as Coll5a1, Lama4, Sparc) have to be made explicit by stating and increase or decrease of their levels more than only a difference. In the future, we will supplement this part of the experiment to improve our research results.

In conclusion, these results could predict potential treatments for patients on a high-fat diet. Semaglutide may protect vascular structure and improve endothelial permeability by reducing the levels of Coll5a1, Lama4, Sparc in extracellular matrix, so as to improve vascular function and achieve vascular protection.

## Data availability statement

The original contributions presented in the study are included in the article/supplementary materials. Further inquiries can be directed to the corresponding author.

## Ethics statement

The animal use protocol for this study has been reviewed and approved by the Animal Ethics Committee of Hebei General Hospital.

## Author contributions

LY and SC conceived and designed the study. LY, QR, SN, and XinC provided materials and samples. LY, QR, SN, XP, XiaC and ZL collected and assembled the data. LY and SC analyzed and interpreted the data. All authors contributed to the article and approved the submitted version.

## Funding

This work was supported by the Hebei Provincial Central leading Local Science and Technology Development funds Project (206Z7702G).

## Conflict of interest

The authors declare that the research was conducted in the absence of any commercial or financial relationships that could be construed as a potential conflict of interest.

## Publisher’s note

All claims expressed in this article are solely those of the authors and do not necessarily represent those of their affiliated organizations, or those of the publisher, the editors and the reviewers. Any product that may be evaluated in this article, or claim that may be made by its manufacturer, is not guaranteed or endorsed by the publisher.

## References

[1] Trends in adult body-mass index in 200 countries from 1975 to 2014: A pooled analysis of 1698 population-based measurement studies with 19·2 million participants. Lancet (2016) 387(10026):1377–96. doi: 10.1016/s0140-6736(16)30054-x PMC7615134 27115820

[2] Piché ME Poirier P Lemieux I Després JP. Overview of epidemiology and contribution of obesity and body fat distribution to cardiovascular disease: An update. Prog Cardiovasc Dis (2018) 61(2):103–13. doi: 10.1016/j.pcad.2018.06.004 29964067

[3] Ma L Ma S He H Yang D Chen X Luo Z. Perivascular fat-mediated vascular dysfunction and remodeling through the AMPK/mTOR pathway in high-fat diet-induced obese rats. Hypertens Res (2010) 33(5):446–53. doi: 10.1038/hr.2010.11 20186150

[4] Liu C Zhou MS Li Y Wang A Chadipiralla K Tian R. Oral nicotine aggravates endothelial dysfunction and vascular inflammation in diet-induced obese rats: Role of macrophage TNFα. PloS One (2017) 12(12):e0188439. doi: 10.1371/journal.pone.0188439 29236702 PMC5728478

[5] Kim ID Cave JW Cho S. Aflibercept, a vegf (vascular endothelial growth factor)-trap, reduces vascular permeability and stroke-induced brain swelling in obese mice. Stroke. (2021) 52(8):2637–48. doi: 10.1161/strokeaha.121.034362 PMC8312568 34192895

[6] Brown E Ozawa K Moccetti F Vinson A Hodovan J Nguyen TA. Arterial platelet adhesion in atherosclerosis-prone arteries of obese, insulin-resistant nonhuman primates. J Am Heart Assoc (2021) 10(9):e019413. doi: 10.1161/jaha.120.019413 33880941 PMC8200741

[7] Soares AG de Carvalho MHC Akamine E. Obesity induces artery-specific alterations: Evaluation of vascular function and inflammatory and smooth muscle phenotypic markers. BioMed Res Int (2017) 2017:5038602. doi: 10.1155/2017/5038602 28466012 PMC5390568

[8] Zhang YY Shi YN Zhu N Zhao TJ Guo YJ Liao DF. Pvat targets vsmcs to regulate vascular remodelling: Angel or demon. J Drug Targeting (2021) 29(5):467–75. doi: 10.1080/1061186x.2020.1859515 33269623

[9] Zhang MJ Zhou Y Chen L Wang YQ Wang X Pi Y. An overview of potential molecular mechanisms involved in vsmc phenotypic modulation. Histochem Cell Biol (2016) 145(2):119–30. doi: 10.1007/s00418-015-1386-3 26708152

[10] Beamish JA He P Kottke-Marchant K Marchant RE. Molecular regulation of contractile smooth muscle cell phenotype: Implications for vascular tissue engineering. Tissue Eng Part B Rev (2010) 16(5):467–91. doi: 10.1089/ten.TEB.2009.0630 PMC2943591 20334504

[11] Bunbupha S Apaijit K Maneesai P Prasarttong P Pakdeechote P. Nobiletin ameliorates high-fat diet-induced vascular and renal changes by reducing inflammation with modulating adipor1 and tgf-β1 expression in rats. Life Sci (2020) 260:118398. doi: 10.1016/j.lfs.2020.118398 32920004

[12] Kapitza C Dahl K Jacobsen JB Axelsen MB Flint A. Effects of semaglutide on beta cell function and glycaemic control in participants with type 2 diabetes: A randomised, double-blind, placebo-controlled trial. Diabetologia. (2017) 60(8):1390–9. doi: 10.1007/s00125-017-4289-0 PMC5491562 28526920

[13] Marso SP Bain SC Consoli A Eliaschewitz FG Jódar E Leiter LA. Semaglutide and cardiovascular outcomes in patients with type 2 diabetes. N Engl J Med (2016) 375(19):1834–44. doi: 10.1056/NEJMoa1607141 27633186

[14] Wang F Chen S Ren L Wang Y Li Z Song T. The effect of silibinin on protein expression profile in white adipose tissue of obese mice. Front Pharmacol (2020) 11:55. doi: 10.3389/fphar.2020.00055 32184719 PMC7059093

[15] Etemad M Christodoulou F Weiss C Klüter H Bugert P. Correlation of clec1b haplotypes with plasma levels of soluble clec-2 in healthy individuals. Platelets. (2021) 32(8):1103–7. doi: 10.1080/09537104.2020.1849601 33251920

[16] Harris JR. Transmission electron microscopy in molecular structural biology: A historical survey. Arch Biochem Biophys (2015) 581:3–18. doi: 10.1016/j.abb.2014.11.011 25475529

[17] Plubell DL Wilmarth PA Zhao Y Fenton AM Minnier J Reddy AP. Extended multiplexing of tandem mass tags (TMT) labeling reveals age and high fat diet specific proteome changes in mouse epididymal adipose tissue. Mol Cell Proteomics. (2017) 16(5):873–90. doi: 10.1074/mcp.M116.065524 PMC5417827 28325852

[18] Jaochico A Sangaraju D Shahidi-Latham SK. A rapid derivatization based lc-ms/ms method for quantitation of short chain fatty acids in human plasma and urine. Bioanalysis. (2019) 11(8):741–53. doi: 10.4155/bio-2018-0241 30993998

[19] Chen L Zhang YH Wang S Zhang Y Huang T Cai YD. Prediction and analysis of essential genes using the enrichments of gene ontology and kegg pathways. PloS One (2017) 12(9):e0184129. doi: 10.1371/journal.pone.0184129 28873455 PMC5584762

[20] O'Connell JD Paulo JA O'Brien JJ Gygi SP. Proteome-wide evaluation of two common protein quantification methods. J Proteome Res (2018) 17(5):1934–42. doi: 10.1021/acs.jproteome.8b00016 PMC5984592 29635916

[21] Pan QR Ren YL Zhu JJ Hu YJ Zheng JS Fan H. Resveratrol increases nephrin and podocin expression and alleviates renal damage in rats fed a high-fat diet. Nutrients. (2014) 6(7):2619–31. doi: 10.3390/nu6072619 PMC4113760 25025298

[22] Wadden TA Bailey TS Billings LK Davies M Frias JP Koroleva A. Effect of subcutaneous semaglutide vs placebo as an adjunct to intensive behavioral therapy on body weight in adults with overweight or obesity: The step 3 randomized clinical trial. Jama. (2021) 325(14):1403–13. doi: 10.1001/jama.2021.1831 PMC7905697 33625476

[23] Pratley R Amod A Hoff ST Kadowaki T Lingvay I Nauck M. Oral semaglutide versus subcutaneous liraglutide and placebo in type 2 diabetes (pioneer 4): A randomised, double-blind, phase 3a trial. Lancet. (2019) 394(10192):39–50. doi: 10.1016/s0140-6736(19)31271-1 31186120

[24] Husain M Birkenfeld AL Donsmark M Dungan K Eliaschewitz FG Franco DR. Oral semaglutide and cardiovascular outcomes in patients with type 2 diabetes. N Engl J Med (2019) 381(9):841–51. doi: 10.1056/NEJMoa1901118 31185157

[25] Ahrén B Masmiquel L Kumar H Sargin M Karsbøl JD Jacobsen SH. Efficacy and safety of once-weekly semaglutide versus once-daily sitagliptin as an add-on to metformin, thiazolidinediones, or both, in patients with type 2 diabetes (sustain 2): A 56-week, double-blind, phase 3a, randomised trial. Lancet Diabetes Endocrinol (2017) 5(5):341–54. doi: 10.1016/s2213-8587(17)30092-x 28385659

[26] Thethi TK Pratley R Meier JJ. Efficacy, safety and cardiovascular outcomes of once-daily oral semaglutide in patients with type 2 diabetes: The pioneer programme. Diabetes Obes Metab (2020) 22(8):1263–77. doi: 10.1111/dom.14054 PMC7384149 32267058

[27] Laurent S Briet M Boutouyrie P. Arterial stiffness as surrogate end point: Needed clinical trials. Hypertension. (2012) 60(2):518–22. doi: 10.1161/hypertensionaha.112.194456 22733473

[28] Jia G Aroor AR DeMarco VG Martinez-Lemus LA Meininger GA Sowers JR. Vascular stiffness in insulin resistance and obesity. Front Physiol (2015) 6:231. doi: 10.3389/fphys.2015.00231 26321962 PMC4536384

[29] Theocharis AD Skandalis SS Gialeli C Karamanos NK. Extracellular matrix structure. Adv Drug Delivery Rev (2016) 97:4–27. doi: 10.1016/j.addr.2015.11.001 26562801

[30] Hong Z Sun Z Li Z Mesquitta WT Trzeciakowski JP Meininger GA. Coordination of fibronectin adhesion with contraction and relaxation in microvascular smooth muscle. Cardiovasc Res (2012) 96(1):73–80. doi: 10.1093/cvr/cvs239 22802110 PMC3584957

[31] Sehgel NL Vatner SF Meininger GA. "Smooth muscle cell stiffness syndrome"-revisiting the structural basis of arterial stiffness. Front Physiol (2015) 6:335. doi: 10.3389/fphys.2015.00335 26635621 PMC4649054

[32] Clementz AG Harris A. Collagen xv: Exploring its structure and role within the tumor microenvironment. Mol Cancer Res (2013) 11(12):1481–6. doi: 10.1158/1541-7786.Mcr-12-0662 PMC4159701 24043668

[33] Tabas I Williams KJ Borén J. Subendothelial lipoprotein retention as the initiating process in atherosclerosis: Update and therapeutic implications. Circulation. (2007) 116(16):1832–44. doi: 10.1161/circulationaha.106.676890 17938300

[34] Durgin BG Cherepanova OA Gomez D Karaoli T Alencar GF Butcher JT. Smooth muscle cell-specific deletion of col15a1 unexpectedly leads to impaired development of advanced atherosclerotic lesions. Am J Physiol Heart Circ Physiol (2017) 312(5):H943–h958. doi: 10.1152/ajpheart.00029.2017 28283548 PMC5451587

[35] Goddi A Carmona A Schroedl L White JM Piron MJ De Leon A. Laminin-α4 is upregulated in both human and murine models of obesity. Front Endocrinol (Lausanne). (2021) 12:698621. doi: 10.3389/fendo.2021.698621 34394003 PMC8355986

[36] Thyboll J Kortesmaa J Cao R Soininen R Wang L Iivanainen A. Deletion of the laminin alpha4 chain leads to impaired microvessel maturation. Mol Cell Biol (2002) 22(4):1194–202. doi: 10.1128/mcb.22.4.1194-1202.2002 PMC134646 11809810

[37] Warren KJ Iwami D Harris DG Bromberg JS Burrell BE. Laminins affect t cell trafficking and allograft fate. J Clin Invest. (2014) 124(5):2204–18. doi: 10.1172/jci73683 PMC4001556 24691446

[38] Li L Song J Chuquisana O Hannocks MJ Loismann S Vogl T. Endothelial basement membrane laminins as an environmental cue in monocyte differentiation to macrophages. Front Immunol (2020) 11:584229. doi: 10.3389/fimmu.2020.584229 33193400 PMC7662115

[39] Butcher EC. Leukocyte-endothelial cell recognition: Three (or more) steps to specificity and diversity. Cell. (1991) 67(6):1033–6. doi: 10.1016/0092-8674(91)90279-8 1760836

[40] Elices MJ Osborn L Takada Y Crouse C Luhowskyj S Hemler ME. Vcam-1 on activated endothelium interacts with the leukocyte integrin vla-4 at a site distinct from the vla-4/fibronectin binding site. Cell. (1990) 60(4):577–84. doi: 10.1016/0092-8674(90)90661-w 1689216

[41] Farrokhi E GhatrehSamani K Hashemzadeh Chaleshtori M Tabatabaiefar MA. Effect of oxidized low density lipoprotein on the expression of runx2 and sparc genes in vascular smooth muscle cells. Iran BioMed J (2015) 19(3):160–4. doi: 10.7508/ibj.2015.03.005 PMC4571011 26025968

[42] Rakipovski G Rolin B Nøhr J Klewe I Frederiksen KS Augustin R. The glp-1 analogs liraglutide and semaglutide reduce atherosclerosis in apoe(-/-) and ldlr(-/-) mice by a mechanism that includes inflammatory pathways. JACC Basic Transl Sci (2018) 3(6):844–57. doi: 10.1016/j.jacbts.2018.09.004 PMC6314963 30623143

